# Exploration of morpholine-thiophene hybrid thiosemicarbazones for the treatment of ureolytic bacterial infections via targeting urease enzyme: Synthesis, biochemical screening and computational analysis

**DOI:** 10.3389/fchem.2024.1403127

**Published:** 2024-05-24

**Authors:** Rubina Munir, Sumera Zaib, Muhammad Zia-ur-Rehman, Hira Javed, Ayesha Roohi, Muhammad Zaheer, Nabiha Fatima, Mashooq Ahmad Bhat, Imtiaz Khan

**Affiliations:** ^1^ Department of Chemistry, Kinnaird College for Women, Lahore, Pakistan; ^2^ Department of Basic and Applied Chemistry, Faculty of Science and Technology, University of Central Punjab, Lahore, Pakistan; ^3^ Applied Chemistry Research Centre, PCSIR Laboratories Complex, Lahore, Pakistan; ^4^ Department of Pharmaceutical Chemistry, College of Pharmacy, King Saud University, Riyadh, Saudi Arabia; ^5^ Department of Chemistry and Manchester Institute of Biotechnology, The University of Manchester, Manchester, United Kingdom

**Keywords:** thiosemicarbazone, thiophene, morpholine, urease, binding interactions, pharmacokinetics

## Abstract

An important component of the pathogenicity of potentially pathogenic bacteria in humans is the urease enzyme. In order to avoid the detrimental impact of ureolytic bacterial infections, the inhibition of urease enzyme appears to be an appealing approach. Therefore, in the current study, morpholine-thiophene hybrid thiosemicarbazone derivatives (**5a-i**) were designed, synthesized and characterized through FTIR, ^1^H NMR, ^13^C NMR spectroscopy and mass spectrometry. A range of substituents including electron-rich, electron-deficient and inductively electron-withdrawing groups on the thiophene ring was successfully tolerated. The synthesized derivatives were evaluated *in vitro* for their potential to inhibit urease enzyme using the indophenol method. The majority of compounds were noticeably more potent than the conventional inhibitor, thiourea. The lead inhibitor, 2-(1-(5-chlorothiophen-2-yl)ethylidene)-*N*-(2-morpholinoethyl)hydrazinecarbothioamide (**5g**) inhibited the urease in an uncompetitive manner with an IC_50_ value of 3.80 ± 1.9 µM. The findings of the docking studies demonstrated that compound **5g** has a strong affinity for the urease active site. Significant docking scores and efficient binding free energies were displayed by the lead inhibitor. Finally, the ADME properties of lead inhibitor (**5g**) suggested the druglikeness behavior with zero violation.

## Graphical Abstract

**Figure:**
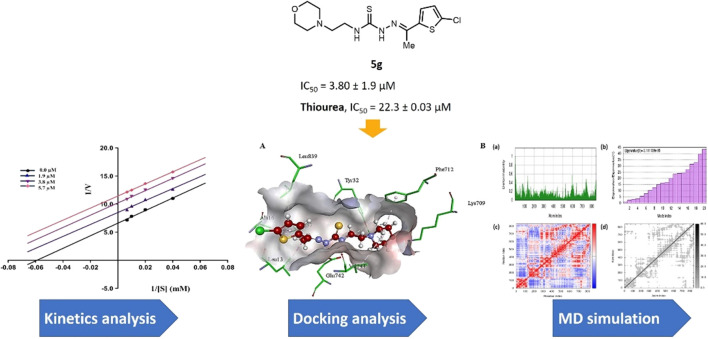
A concise library of morpholine-thiophene hybrid thiosemicarbazones was synthesized for the identification of potent inhibitors of urease enzyme to treat ureolytic bacterial infections.

## 1 Introduction

Urease (amidohydrolase and phosphotriesterase; EC 3.5.1.5) is a metallopeptidase enzyme extensively found in prokaryotes as well as some eukaryotes and catalyzes the conversion of urea to ammonia and carbamate (Xiao et al., 2013; Mobley and Hausinger, 1989; Konieczna et al., 2012; D’Agostino and Carradori, 2024). Urease plays an important role in enzymology and speeds up the catalytic activity by 10^14^ times. The active site of urease contains three water molecules and two Ni atoms connected through a hydroxide bond (Mobley and Hausinger, 1989). The urease from *H. pylori* (*Helicobacter pylori*) is the most commonly investigated bacterial urease due to its infectious association with various diseases like gastritis, stomach cancer, and peptic ulcer (Kafarski and Talma, 2018; Hameed et al., 2019). The potential of the bacteria to produce ammonia which nullifies the tough acidic environment around them allows these bacteria to thrive in the acidic environment of the stomach (Zambelli et al., 2011). Urease is a key target for the design of drugs against *H. pylori*, therefore, urease inhibition treatments are considered to be a potentially effective way to treat disorders caused by urease-producing bacteria (Kafarski and Talma, 2018). The scientific community has paid a close attention towards the discovery of urease inhibitors because of their potential application against *H. pylori* urease. Several urease inhibitors have been identified until now, but only a limited number have progressed to the latter phases of drug development (Ibrar et al., 2013; Svane et al., 2020). These inhibitors include oxadiazoles (Mentese et al., 2017), coumarins (Ayaz et al., 2006), hydrazides (Amtul et al., 2002), pyrimidines (Rauf et al., 2012), triazoles (Mentese et al., 2017), amides (Rauf et al., 2016), triazolothiadiazoles (Hanif et al., 2012), thioureas (Khan et al., 2006), thiosemicarbazides (Rego et al., 2018a), phosphoramidates (Kot et al., 2001), hydroxamic acids (Muri et al., 2003), flavonoid glycosides (Babu et al., 2017), Schiff bases, and many others (Abid et al., 2010; Kazmi et al., 2019; Yang et al., 2023; Al-Fakhrany and Elekhnawy, 2023; Sepehri and Khedmati, 2023; Al-Rooqi et al., 2023; Khan et al., 2024; Akash et al., 2024; Viana et al., 2024; Wang et al., 2024; Valenzuela-Hormazabal, et al., 2024; Saeed K. et al., 2024; Ullah et al., 2024].

In parallel, thiosemicarbazone is a sulfur-nitrogen donor ligand which serves as a robust precursor to heterocyclic compounds (Castro et al., 2023; Palakkeezhillam et al., 2023). Thiosemicarbazones are synthesized by the condensation reaction of thiosemicarbazide with suitable aldehydes or ketones (Devi et al., 2022). The presence of amide, imine and thione groups make them potential polydentate ligands (Yousef and El-Reash, 2020). In 1960s, the first biological application of thiosemicarbazones was found against tuberculosis and leprosy (Volynets et al., 2019). Heterocyclic thiosemicarbazones have the ability to diffuse through semipermeable membrane into the cell lining (Nkungli et al., 2023). These are known for chelating property towards metals like nickel, zinc, cadmium, cobalt, copper and show a wide range of applications in analytical chemistry, pharmacological chemistry and nuclear medicine (Dilworth and Hueting, 2012; Raicopol et al., 2020). Various studies have demonstrated that the biological and chemical activities of thiosemicarbazones and their complexes may be tuned by altering the metal center attached to sulfur and/or hydrazine as well as the substituent attached to amide nitrogen (Summers, 2019). In recent years, thiosemicarbazone derivatives have been documented for their profound biological activities including antidiabetic (Basri et al., 2023), anticancer (Sibuh et al., 2021; Bai et al., 2022), antibacterial (Hassan et al., 2020), antifungal (Bajaj et al., 2021), anti-inflammatory (Saeed A. et al., 2024), anti-Alzheimer’s (Khan et al., 2023; Jalil et al., 2024), anti-melanoma (Kurt et al., 2024), antitubercular (Gobis et al., 2022) and antioxidant (Yang et al., 2020) activities. The diverse variety of biological potential shown by this class of compounds is possibly due to the multifunctional nature of thiosemicarbazone motif which carries C=S and NH electron donor groups as well as the hydrophobic aryl substituent (Pati et al., 2018). The structural similarity of thiosemicarbazide scaffold with thiourea makes them an ideal candidate for urease inhibitory investigation (Islam et al., 2019; Islam et al., 2023).

**FIGURE 1:**
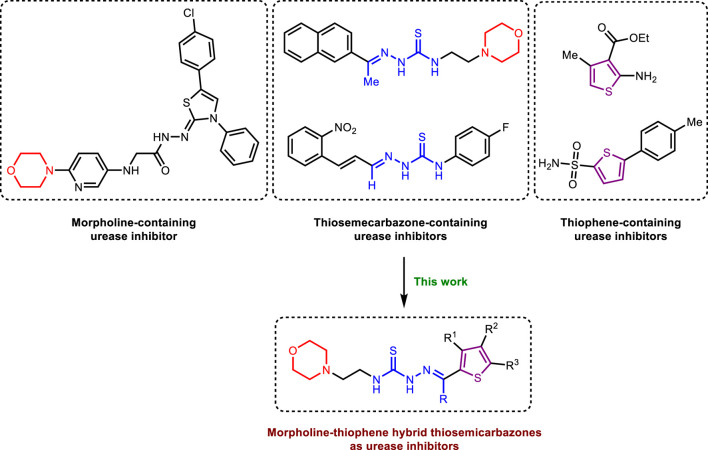
Literature reported urease inhibitors and rationale for strategic development of morpholine-thiophene hybrid thiosemicarbazones.

In parallel, morpholine heterocycle has been ranked as an important pharmacophore encountered in numerous drugs used for the treatment of bacterial infections and other diseases (Bektaş et al., 2013; Bektaş et al., 2017; Kumari and Singh, 2020; Sıcak et al., 2023). The incorporation of morpholine ring into various drug molecules leads to enhanced potency through the formation of molecular interactions with the target protein or by modulating the pharmacokinetic profile. The World Drug Index has declared more than 100 drugs featuring morpholine ring (Kumari and Singh, 2020). Thiophene containing compounds, on the other hand, have also been reported to exhibit potent inhibitory efficacy against urease (Khan et al., 2010; Noreen et al., 2017; Akyüz et al., 2022; Güven et al., 2023). Both morpholine and thiophene rings have been precedented in literature showing significant urease inhibitory efficacy (Khan et al., 2010; Bektaş et al., 2013; Bektaş et al., 2017; Rego et al., 2018b; Akyüz et al., 2022). Similarly, thiosemicarbazones bearing diverse structural features have also been documented as potent anti-urease agents (Islam et al., 2019; Islam et al., 2023). In view of the anti-urease potential of all three distinct pharmacophores, we aimed to explore their combined urease inhibitory effect as a single entity to successfully deliver the lead drug candidates (Figure 1). Therefore, the structural optimization led us to design and synthesize a concise series of morpholine- and thiophene-containing thiosemicarbazone conjugates which were evaluated against urease enzyme. The obtained *in vitro* inhibitory data was reinforced with the molecular docking analysis elaborating the ligand-enzyme interaction within the active site of urease enzyme. Molecular dynamics simulations as well as ADMET properties were also computed.

## 2 Results and discussion

### 2.1 Synthetic chemistry

**SCHEME 1:**
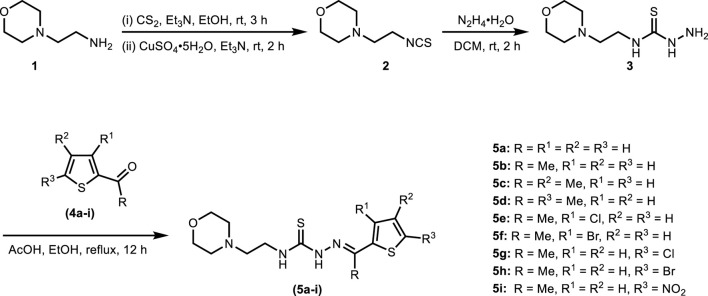
Synthetic layout for morpholine-thiophene hybrid thiosemicarbazones.

The synthesis of title thiosemicarbazones was achieved in a facile manner by following the route illustrated in Scheme 1. Base-catalyzed reaction of 4-(2-aminoethyl)morpholine (**1**) with carbon disulfide followed by desulfurization using copper(II) sulfate provided access to 4-(2-isothiocyanatoethyl)morpholine (**2**) (Mandapati et al., 2017). Subsequently, hydrazination of isothiocyanate (**2**) with hydrazine monohydrate at room temperature furnished *N*-(2-morpholinoethyl)hydrazinecarbothioamide (**3**) which was condensed in the final step with (un)substituted formyl/acetylthiophenes (**4a-i**) to afford thiosemicarbazones (**5a-i**) in good to excellent isolated yields.

The FTIR spectra of the synthesized thiosemicarbazones displayed N-H stretching bands at 3,345–3,312 cm^−1^, C=N around 1,530–1,506 cm^−1^ and C=S stretching vibrations at 1,142–1,102 cm^−1^. ^1^H NMR spectra exhibited two peaks concerning the secondary thioamide protons referring to = N-NH and C-NH. The = N-NH signal appeared as a singlet peak downfield between 10.45 and 11.57 ppm. The C-NH proton splits into a triplet due to its spin-spin coupling with the neighbouring methylene protons and was observed around 8.15 ppm. Hydrogens of morpholine ring gave two sets of triplets near 2.44 ppm and 3.60 ppm, the later signal though merged with the peak of the methylene in most of the compounds. The thiophene ring protons resonated between 6.78 and 8.09 ppm depending upon the substituent on the ring.

^13^C NMR spectral data endorsed the structures of the synthesized compounds where C=S functional group appeared at 178 ppm. Signals of morpholine ring carbon atoms resonated at 53.5 and 66.7 ppm while resonances for linear methylene carbon atoms were observed at 40.4 and 56.5 ppm. Thiophene ring carbon atoms resonated between 108.9 and 178.2 ppm. Likewise, the elemental analyses and mass spectral data of all the derivatives (**5a-i**) were in agreement with the anticipated structures.

### 2.2 *In vitro* urease inhibition and structure-activity relationship analysis

The synthesized morpholine-thiophene hybrid thiosemicarbazones (**5a-i**) were tested to determine their urease inhibitory effect. In comparison to the standard inhibitor, thiourea, which showed inhibitory activity with an IC_50_ value of 22.31 ± 0.03 µM, these compounds were discovered to be effective inhibitors of the urease demonstrating outstanding inhibition with IC_50_ in the range of 3.80–5.77 µM. The inhibitory results of the tested compounds are shown in Table 1.

**TABLE 1:** *In vitro* urease inhibitory activity of target compounds (**5a-i**).

Compound	Structure	Urease inhibition^[a]^ IC_50_ ± SEM µM
**5a**	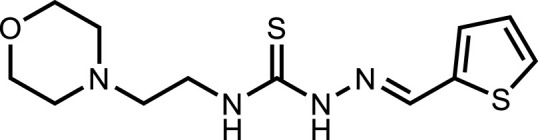	4.94 ± 2.7
**5b**	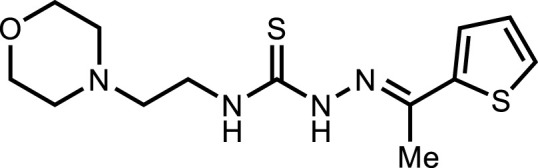	4.96 ± 3.0
**5c**	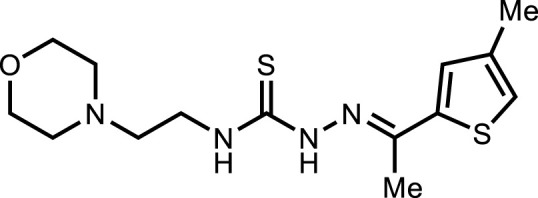	4.00 ± 2.4
**5d**	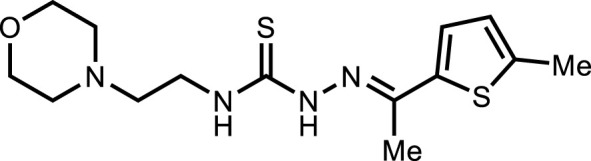	4.60 ± 2.6
**5e**	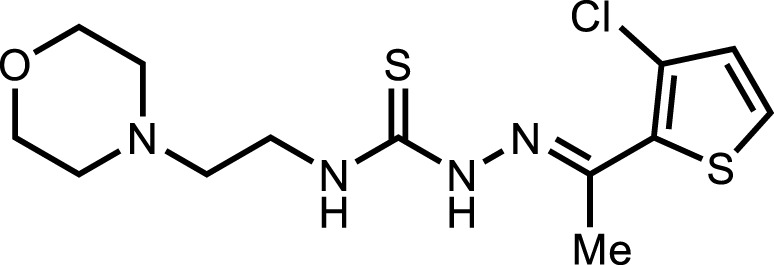	4.81 ± 1.5
**5f**	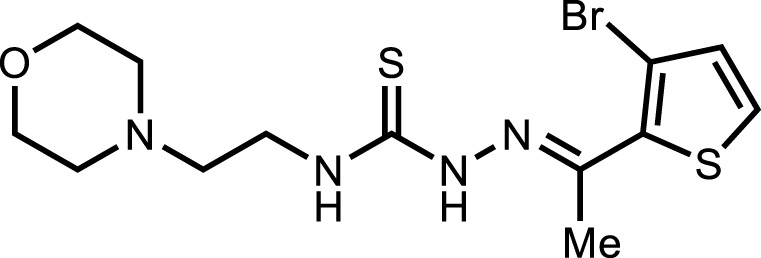	5.77 ± 0.7
**5g**	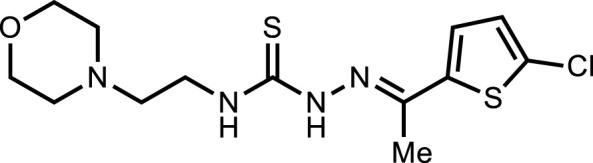	3.80 ± 1.9
**5h**	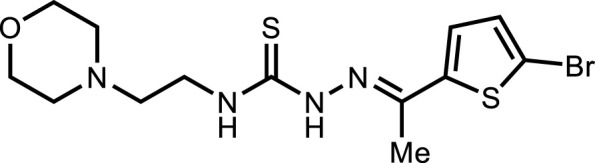	3.98 ± 2.2
**5i**	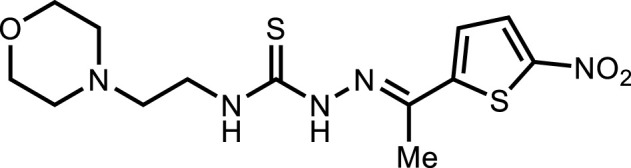	3.90 ± 2.7
**Thiourea**	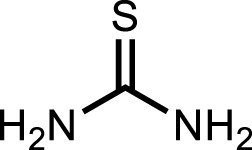	22.3 ± 0.03

^[a]^All experiments were performed in triplicate.

As depicted in Table 1, a varied degree of inhibitory potential was observed for tested compounds (**5a-i**). All the compounds showed remarkable inhibitory potential identifying compound **5g** as the lead inhibitor exhibiting strong inhibitory potential with an IC_50_ value of 3.80 ± 1.9 µM, more than the standard inhibitor.

*In vitro* analysis of compounds against urease showed that compound **5a** ((*E*)-*N*-(2-morpholinoethyl)-2-(thiophen-2-ylmethylene)hydrazinecarbothioamide) exhibited remarkable inhibitory potential with an IC_50_ value of 4.94 ± 2.7 µM, 4.5-fold strong inhibitory potential than thiourea. Similar inhibition potential was observed for compound **5b** where an additional methyl group was present at the imine moiety. Compound **5b** demonstrated an IC_50_ value of 4.96 ± 3.0 µM. However, the addition of methyl substituent at the thiophene ring on position 4 (**5c**) showed inhibitory potential with an IC_50_ value of 4.00 ± 2.4 µM. Switching the position of methyl substituent at thiophene ring from 4 to 5 (**5d**) exhibited slightly lower inhibitory potential than **5c** but the strength was still 4.8-fold better than thiourea. Furthermore, the replacement of methyl substituent with a chloro group at thiophene ring (position 3) demonstrated similar inhibition results with an IC_50_ value of 4.81 ± 1.5 µM as shown by compound **5e**. In compound **5f**, the replacement of chloro with bromo demonstrated the least potential among all compounds (**5a-i**) with an IC_50_ value of 5.77 ± 0.7 µM. However, the inhibitory strength was still stronger than thiourea. Compound **5g** exhibited inhibitory potential with the lowest IC_50_ value. The presence of the chloro group at the fifth position of thiophene ring demonstrated the highest inhibitory strength with an IC_50_ value of 3.80 ± 1.9 µM. The presence of other electron-withdrawing groups (Br, NO_2_) at the same position also showed remarkable inhibition of urease with IC_50_ values of 3.98 ± 2.2 and 3.90 ± 2.7 µM, respectively, as depicted by compounds **5h** and **5i**. In general, all the tested compounds exhibited several folds higher inhibitory power compared to thiourea, standard inhibitor. Although a varied degree of inhibition was noticed among the tested series of thiosemicarbazones, the extent of biological efficacy was found least dependent on the type of substituent on thiophene ring. Therefore, considering the incorporation of further ring variations as well as changing the ethylmorpholine unit on the left hand side of the synthesized molecules could exert beneficial role in designing new families of thiosemicarbazones for potent urease inhibitory properties.

### 2.3 Kinetics analysis

**FIGURE 2:**
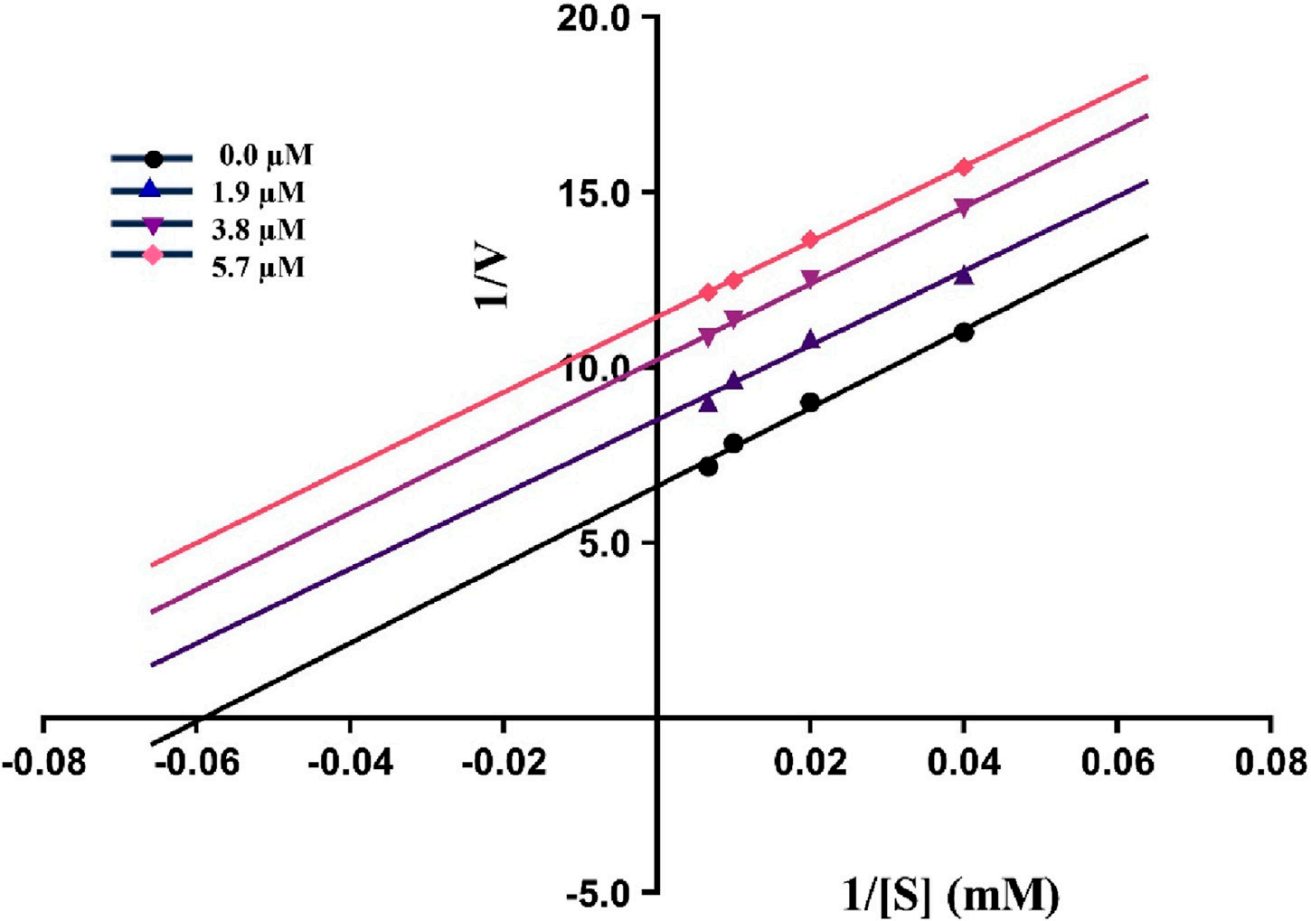
The Lineweaver-Burk plot of **5g** at various substrate (urea) and inhibitor concentrations showing uncompetitive inhibition as indicated by parallel slopes.

The kinetics experiments of the most potent inhibitor **5g** having the least IC_50_ value were performed and the results obtained were utilized to construct Lineweaver-Burk plot using GraphPad Prism version 10.2.1. These experiments helped in the assessment of K_*m*_ and V_*max*_ values to predict the mechanism of urease inhibition by plotting reciprocal of reaction rate 1/V (*y*-axis) against reciprocal of substrate concentrations 1/[S] (*x*-axis). As illustrated in Figure 2, compound **5g** inhibits urease in an uncompetitive manner as all the slopes (K_*m*_/V_*max*_) are parallel to each other at different inhibitor concentrations. These results indicate that both the V_*max*_ and K_*m*_ decrease as the concentration of inhibitor increases.

### 2.4 Docking analysis

The lead compound exhibiting the best inhibitory potential with low IC_50_ value was docked within the jack bean urease active site. Similar to literature examples (Elbastawesy et al., 2021; Hina et al., 2023), PDB ID: 3LA4 was selected as a computational urease model to perform the docking studies for compounds investigated in this research. Compound **5g** demonstrated binding affinity in micromolar range along with various interactions with the active site amino acid residues, as shown in Table 2.

**TABLE 2:** The binding interactions between receptor residues and potent inhibitor **5g**.

Compound (g)	Binding interactions			
	Ligand atom	Receptor residue	Interaction type	Distance (Å)
**5**	N4	Tyr32	C-H bond	3.08
	H32	Tyr32	C-H bond	2.95
	H23	Tyr32	C-H bond	2.80
	O1	Lys709	C-H bond	2.88
	H35	Glu742	C-H bond	2.26
	H34	Glu742	C-H bond	2.13
	Thiophene ring	Leu839	π-alkyl	5.14
	C20	Leu13	π-alkyl	4.98
	C20	Ala16	alkyl	4.24
	C20	Ala37	alkyl	4.15
	Morpholine ring	Val744	alkyl	5.19
	Thiophene ring	Ala16	π-alkyl	4.29
	Morpholine ring	Phe712	π-alkyl	5.34

**FIGURE 3:**
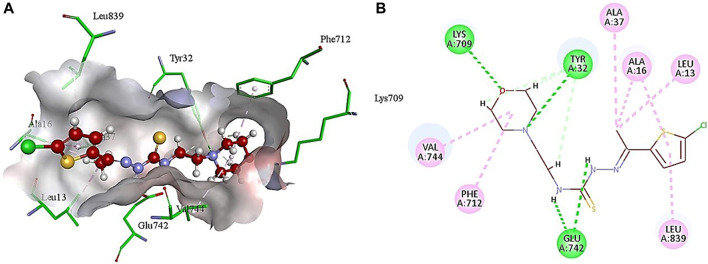
Illustration of 3D **(A)** and 2D **(B)** interactions of compound **5g** with urease.

The analysis of intermolecular interactions of potent inhibitor **5g** within the binding pocket of urease revealed that several amino acids including Leu13, Ala16, Tyr32, Ala37, Lys709, Phe712, Glu742, Val744 and Leu839 are significantly involved in the formation of key contacts. Compound **5g** showed conventional hydrogen bonding with Tyr32, Glu742 and Lys709 present at a distance of 3.08, 2.26 and 2.88 Å, respectively. However, carbon hydrogen bond interactions were formed by the morpholine ring of **5g** with Tyr32 (2.79, 2.80, 2.95 Å). Additionally, the morpholine ring of **5g** also developed alkyl and π-alkyl interactions with Val744 (5.19 Å) and Phe717 (5.34 Å), respectively. Amino acid residues such as Ala16 and Leu839 interact with the thiophene ring via π-alkyl interactions having bond lengths of 4.29 and 5.14 Å, respectively. On the other hand, methyl group of **5g** showed alkyl and π-alkyl interactions with Leu13 (4.98 Å), Ala16 (4.24 Å) and Ala37 (4.15 Å), as shown in Figure 3.

The other compounds were also docked in the same active site, and the sequence of the decrease in estimated affinity (**5g** > **5i** > **5h** > **5c** > **5d**> **5e** > **5a**>**5b** > **5f** > **positive control**) verifies the IC_50_ values obtained in an *in vitro* assay against urease (Supplementary Figure S1).

The conventional inhibitor (thiourea) showed only three interactions (hydrogen bonds) with two amino acid residues (Ser421 and Thr715) when docked in the same binding pocket of urease. Therefore, it depicts more IC_50_ value as compared to morpholine-thiophene hybrid thiosemicarbazone derivatives. Moreover, all the synthesized morpholine-thiophene hybrid thiosemicarbazones (**5a-i**) showed maximum intermolecular interactions with the active site residues of urease. That is why, their IC_50_ values have no major differences. The thiophene ring as the main attachment with various substituents interacts with urease active site residues via alkyl, π-alkyl, π-anion, and π-cation interactions as shown in Supplementary Figure S2.

The presence of methyl substituent on the thiophene ring (**5c** and **5d**) facilitates the formation of alkyl interaction. On the other hand, the bromo substituent on thiophene ring in **5f** and **5h** participates in the formation of alkyl interaction with amino acid residues of urease. In addition, nitro substitution on thiophene ring enables π-cation, π-anion and attractive charges interactions. The presence of chloro group as a substituent on the thiophene ring in **5e** and **5g** has not shown any interaction with the amino acid residues of the binding site. Apart from these, hydrogen bond interactions were observed as the common contacts within all compounds docked with the binding site residues of urease, but the number of these interactions varied as **5g** showed 7 H-bond interactions. The presence of more hydrogen bond interactions makes **5g** the most potent and lead inhibitor showing the lowest IC_50_ value of 3.80 ± 1.9 µM.

These findings also correlate with the literature precedent (Hassan and Švajdlenka, 2017) where hydrogen bonding involving amino acid residues Tyr32 and Lys709 are mainly responsible for the anti-urease activity. Another similar report (Kataria and Khatkar, 2019) also revealed that compounds having the best antibacterial activity develop key interactions with the same amino acids (Val744, Lys709, Try32, Ala16, Ala37, Glu742, Leu839, and Leu13) as mentioned in Figure 3. However, the computational analysis of standard inhibitor (thiourea) does not match with the literature example (Saeed et al., 2013). Regardless of this study, several other reports showed the inhibition of urease by various compounds thus making crucial interactions with the same amino acids (Hassan and Švajdlenka, 2017; Kataria and Khatkar, 2019; Hina et al., 2023), as mentioned in Figure 3. These results showed that compound **5g** has the potential to exhibit antibacterial activity by the inhibition of urease.

### 2.5 SeeSAR visual drug design

**FIGURE 4:**
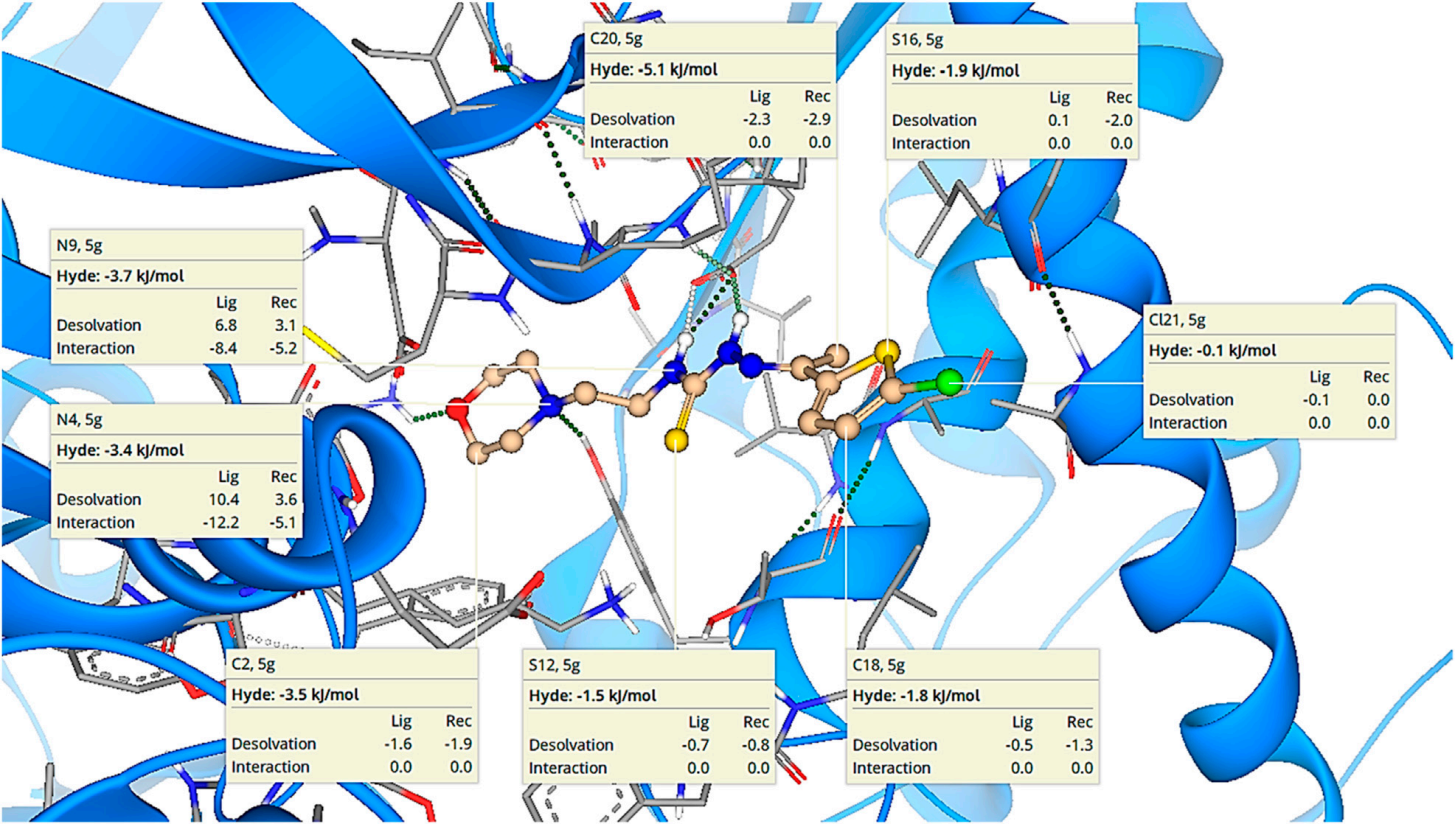
Visual representation of HYDE of compound **5g** showing the favorable atoms involved in affinity inside the active site of urease (PDB: 3LA4).

LeadIT software was used to evaluate the Hydrogen bond and DEhydration energy (HYDE) of potent inhibitor **5g**. HYDE studies showed that the lead inhibitor (**5g**) has the highest affinity towards urease. Figure 4 demonstrates the HYDE of atoms of compound **5g** indicating their involvement in the binding affinity. The lower the HYDE of an atom, the higher is its involvement in the estimated affinity.

### 2.6 ADMET properties

The ADMET properties of compound **5g** were determined via calculating various parameters, including physicochemical properties, lipophilicity, water solubility, pharmacokinetics, druglikeness, and medicinal chemistry. These properties determine the likelihood of a drug. In our case, these properties indicated that compound **5g** is the lead inhibitor with 0 violation, as shown in Table 3. The pharmacokinetic properties and the lowest IC_50_ value support compound **5g** to serve as a lead inhibitor. Furthermore, the comparative analysis of pharmacokinetic parameters of **5g** from four different software (SwissADME, pkCSM, preADMET and vNN-ADMET) is shown in Table 4 (Dulsat et al., 2023). These results interpreted that compound **5g** can be absorbed from gastrointestinal tract but unable to cross the barrier between blood and brain tissues. In addition, compound does not inhibit the P-glycoprotein (P-gp) but may act as a substrate of P-gp. The in depth toxicological analysis of **5g** using admetSAR was also obtained in the form of probability score (Table 5). The prediction having probability of 0.7 or higher are more significant and included in the analysis of **5g**. According to this, **5g** exhibits non-carcinogenic and non-nephrotoxic character, whereas, it can induce respiratory and reproductive toxicity. Moreover, the compound does not cause any irritation to eyes or integumentary lining.

**TABLE 3:** ADME properties of compound **5g** from SwissADME.

Properties	Predictions
Physiochemical properties	
Formula	C_13_H_19_ClN_4_OS_2_
Molecular weight (g/mol)	346.90
No. of heavy atoms	21
No. of aromatic heavy atoms	5
Fraction C_sp3_	0.54
No. of rotatable bonds	7
No. of H-bond acceptors	3
No. of H-bond donors	2
Molar refractivity	95.61
TPSA (Å^2^)	109.22
Lipophilicity	
Log *Po/w* (iLOGP)	3.15
Log *Po/w* (XLOGP3)	2.46
Log *Po/w* (WLOGP)	1.54
Log *Po/w* (MLOGP)	1.07
Log *Po/w* (SILICOS-IT)	4.26
Consensus Log *Po/w*	2.50
Water solubility	
Log S (ESOL)	−3.25
Solubility	1.93e-01 mg/mL; 5.56e-04 mol/L
Class	Soluble
Log S (Ali)	−4.40
Solubility	1.39e-02 mg/mL; 4.00e-05 mol/L
Class	Moderately soluble
Log S (SILICOS-IT)	−3.98
Solubility	3.66e-02 mg/mL; 1.05e-04 mol/L
Class	Soluble
Druglikeness	
Lipinski	Yes
Ghose	Yes
Veber	Yes
Egan	Yes
Muegge	Yes
Bioavailability Score	0.55
Medicinal chemistry	
PAINS	0 alert
Brenk	2 alerts: imine_1, thiocarbonyl_group
Leadlikeness	Yes
Synthetic accessibility	3.38

**TABLE 4:** Pharmacokinetics properties of **5g** using different software.

Properties	Predictions			
	SwissADME	pkCSM	PreADMET	vNN-ADMET
GI absorption	High	92.285%	95.213%	-
BBB permeability	No	−0.013	No	No
P-gp substrate	No	Yes	-	Yes
P-gp inhibitor	-	No	No	No
CYP1A2 inhibitor	Yes	No	-	No
CYP2C19 inhibitor	Yes	No	No	No
CYP2C9 inhibitor	No	No	No	No
CYP2D6 inhibitor	No	No	No	No
CYP3A4 inhibitor	Yes	Yes	No	No

**TABLE 5:** Toxicological evaluation of **5g** using admetSAR.

Properties	Predictions
Carcinogenicity	No (0.7700)
Eye corrosion	No (0.9768)
Eye irritation	No (0.9854)
Skin irritation	No (0.7451)
Skin corrosion	No (0.8908)
Micronuclear	Yes (0.7300)
Respiratory toxicity	Yes (0.8889)
Reproductive toxicity	Yes (0.9000)
Mitochondrial toxicity	Yes (0.9500)
Nephrotoxicity	No (0.8144)
Androgen receptor binding	No (0.7346)

Biotransformation 3.0 showed the reactive potential of **5g** with bacterial enzyme UDP-glucuronosyltransferase (Table 6). This enzyme can biotransform compound **5g** into another compound via N-glucuronidation of tertiary aliphatic amine. The same software was used to access the abiotic transformation of **5g** and it was disclosed that compound could undergo reduction, ozonation and chlorination under abiotic conditions. Xenosite analysis interpreted that **5g** can undergo epoxidation, N-dealkylation, and have reactive site to bind with the DNA (Table 6). The predictions of Xenosite are scored on the basis of color such as red color scored for 1, yellow between 1 and 0.5, green color for 0.5, blue between 0.5 and zero and white for zero. The higher the score, the greater will be the chance of that site to be reactive (Dang et al., 2020).

**TABLE 6:** Biotransformation and XenoSite prediction of **5g**.

Biotransformation	
Bacterial UDP-glucuronosyltransferase	Substrate
XenoSite prediction	
Epoxidation	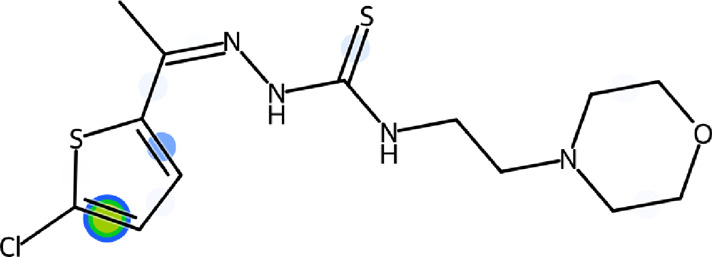
N-dealkylation	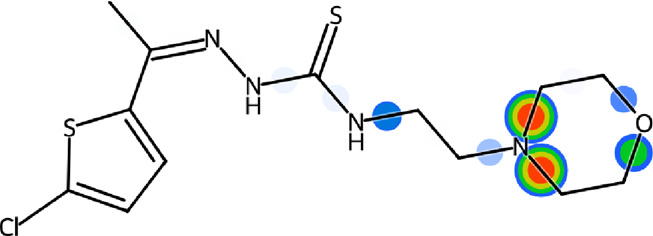
Reactivity with DNA	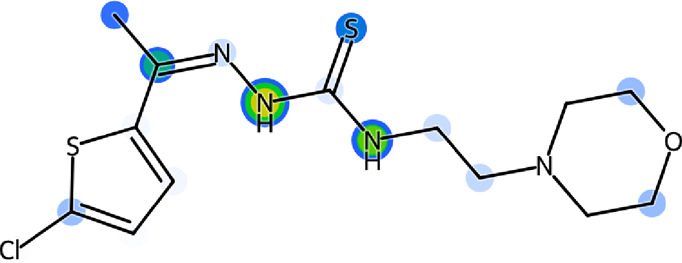

### 2.7 Molecular dynamics simulation

**FIGURE 5:**
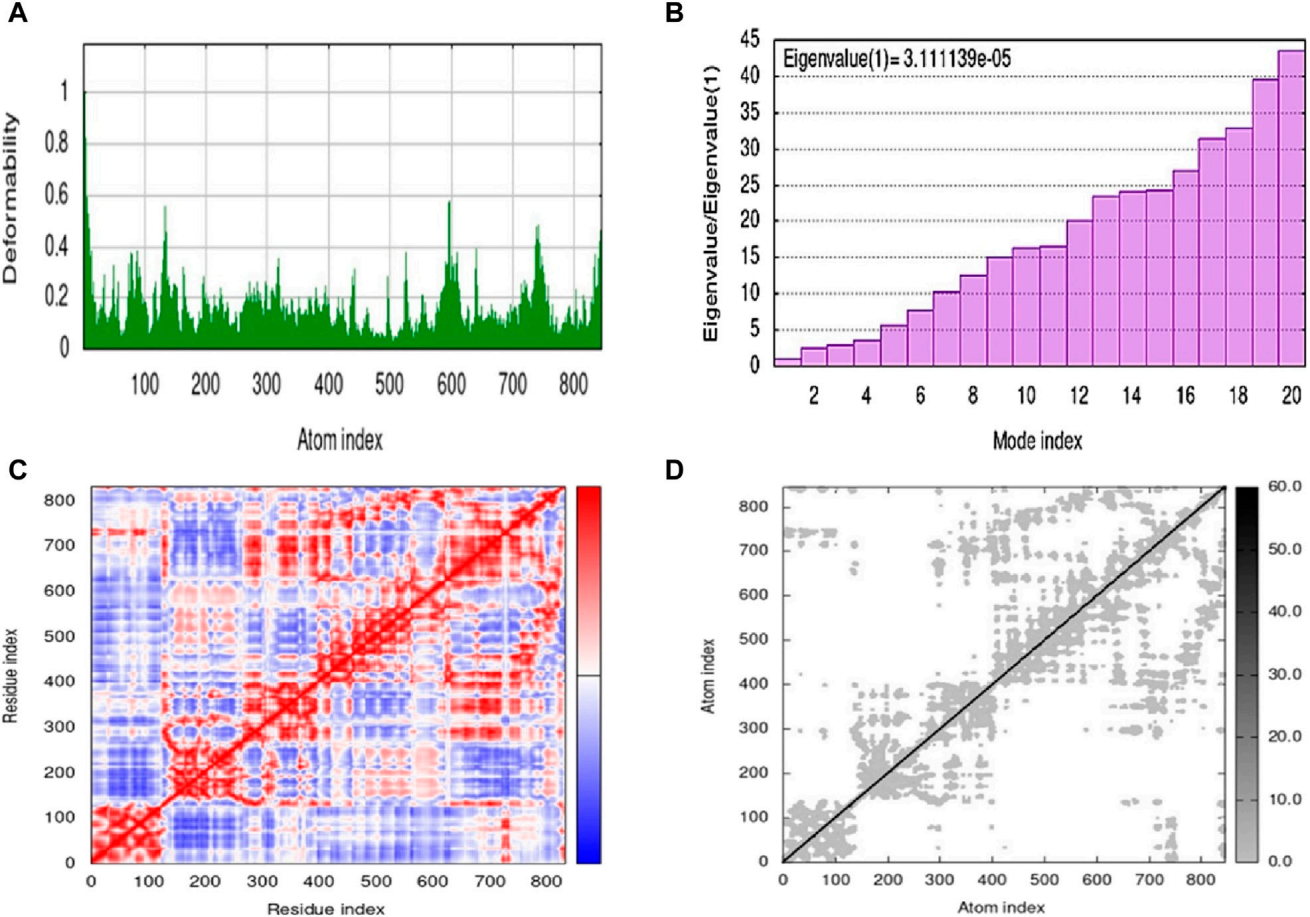
Molecular dynamics simulation of 5g by iMOD. **(A)** deformability plot; **(B)** eigenvalue; **(C)** covariance map; and **(D)** elastic network model.

The molecular dynamics simulation was performed by using an iMOD server. This online server is used for the estimation of the stability of the ligand-protein complex. The results of molecular dynamics simulation of **5g** are shown in Figure 5. The lower peaks are associated with lower deformability, while higher peaks represent higher deformability, as shown in Figure 5A. However, the eigenvalue represents the energy required for the deformation of the structure with an inverse relation, as shown in Figure 5B. Moreover, three different colors represent the covariance, such as correlated as red, non-correlated as blue, and anti-correlated as white (Figure 5C). Figure 5D represents the elastic model network that is associated with spring formation. The gray color indicates the extent of spring formation, and most of the atoms are forming spring.

## 3 Materials and methods

### 3.1 Chemicals and instrumentation

The chemicals and solvents used to perform synthetic chemistry were of analytical grade and obtained from local dealers of Merck, Fisher and Fluka. Thin layer chromatography was carried out on aluminum plates coated with silica gel 60 F_254_ (Merck) in an appropriate eluting system and UV lamp was employed for visualization of spots. Melting points were recorded in open capillaries on Gallenkamp melting point apparatus and are uncorrected. ^1^H NMR spectra were recorded in DMSO-*d*_*6*_ on Bruker Avance NMR spectrometer at 300 MHz while ^13^C NMR at 75 MHz. Chemical shifts are reported as *δ* values in parts per million (ppm) compared to TMS as internal standard or the residual deuterated solvent used. Coupling constant (*J*) is given in Hertz. FTIR spectra (neat) were recorded on Bruker FTIR spectrophotometer. Mass spectra were obtained using LCMS 6495c Agilent whereas elemental analysis was attained on LECO 630–200-200 TRUSPEC CHNS micro analyzer and the experimental values are within ±0.4% of the calculated values.

Synthesis of compounds **2** and **3** was achieved by following the reported synthetic procedures (Mandapati et al., 2017; He et al., 2019).

### 3.2 General procedure for the synthesis of thiosemicarbazones (5a-i)

To a stirred solution of *N*-(2-morpholinoethyl)hydrazinecarbothioamide **3** (1 mmol) in absolute ethanol (20 mL) was added thiophene-2-carbaldehyde (or un/substituted acetylthiophene) **4** (1 mmol) and two to three drops of glacial acetic acid. The reaction mixture was refluxed for 10–12 h and then allowed to stand for 48 h in a refrigerator. The precipitated crude product was filtered and recrystallized from methanol to give thiosemicarbazones (**5a-i**).

#### 3.2.1 *N*-(2-morpholinoethyl)-2-(thiophen-2-ylmethylene)hydrazinecarbothioamide (5a)

Yield 79%. Light brown crystalline solid. Mp 136°C–138 °C. FT-IR (ῡ, cm^−1^): 3,345 (N-H), 3,146 (Ar-H), 2,991 (C-H), 1,525 (C=N), 1,142 (C=S); ^1^H NMR (DMSO-*d*_*6*_, 300 MHz) *δ* 2.44 (t, *J* = 4.2 Hz), 2.53 (t, *J* = 6.3 Hz), 3.62–3.65 (m, 6H), 7.12 (t, *J* = 4.2 Hz, 1H), 7.44 (d, *J* = 3.3 Hz, 1H), 7.68 (d, *J* = 5.1 Hz, 1H), 8.14 (t, *J* = 4.8 Hz, 1H), 8.26 (s, 1H), 11.57 (s, 1H); ^13^C NMR (DMSO-*d*_*6*_, 75 MHz) *δ* 40.4, 53.5, 56.5, 66.8, 128.5, 129.2, 131.3, 137.7, 139.2, 176.8; Anal. Calcd. for C_12_H_18_N_4_OS_2_: C, 48.30; H, 6.08; N, 18.77; S, 21.49%; Found: C, 48.48; H, 6.12; N, 18.85; S, 21.63%.

#### 3.2.2 *N*-(2-morpholinoethyl)-2-(1-(thiophen-2-yl)ethylidene)hydrazinecarbothioamide (5b)

Yield 93%. Yellow crystalline solid. Mp 142°C–144 °C. FT-IR (ῡ, cm^−1^): 3,326 (N-H), 3,160 (Ar-H), 2,968 (C-H), 1,521 (C=N), 1,102 (C=S); ^1^H NMR (DMSO-*d*_*6*_, 300 MHz) *δ* 2.34 (s, 3H), 2.44 (t, *J* = 3.9 Hz, 4H), 2.53 (t, *J* = 6.3 Hz, 2H), 3.63–3.67 (m, 6H), 7.09 (d, *J* = 4.5 Hz, 1H), 7.50 (t, *J* = 3.9 Hz, 1H), 7.63 (dd, *J* = 5.1, 0.9 Hz, 1H), 8.16 (t, *J* = 4.8 Hz, 1H), 10.47 (s, 1H); ^13^C NMR (DMSO-*d*_*6*_, 75 MHz) *δ* 15.1, 40.4, 53.5, 56.5, 66.8, 128.3, 128.6, 129.0, 143.4, 145.1, 177.7; LCMS m/z [M + H]^+^: 313.50; Anal. Calcd. for C_13_H_20_N_4_OS_2_: C, 49.97; H, 6.45; N, 17.93; S, 20.52%; Found: C, 50.09; H, 6.56; N, 18.01; S, 20.60%.

#### 3.2.3 2-(1-(4-Methylthiophen-2-yl)ethylidene)-*N*-(2-morpholinoethyl)hydrazinecarbothioamide (5c)

Yield 85%. Yellow crystalline solid. Mp 112°C–114 °C. FT-IR (ῡ, cm^−1^): 3,319 (N-H), 3,179 (Ar-H), 2,961 (C-H), 1,506 (C=N), 1,139 (C=S); ^1^H NMR (DMSO-*d*_*6*_, 300 MHz) *δ* 2.20 (s, 3H), 2.30 (s, 3H), 2.44 (t, *J* = 3.9 Hz, 4H), 2.53 (t, *J* = 6.3 Hz, 2H), 3.62–3.65 (m, 6H), 7.20 (t, *J* = 0.9 Hz, 1H), 7.33 (d, *J* = 1.2 Hz, 1H), 8.15 (t, *J* = 4.8 Hz, 1H), 10.45 (s, 1H); ^13^C NMR (DMSO-*d*_*6*_, 75 MHz) *δ* 15.0, 15.9, 40.4, 53.5, 56.5, 66.8, 124.2, 130.6, 138.1, 143.0, 144.9, 177.6; LCMS m/z [M + H]^+^: 327.50; Anal. Calcd. for C_14_H_22_N_4_OS_2_: C, 51.50; H, 6.79; N, 17.16; S, 19.64%; Found: C, 51.54; H, 6.87; N, 17.30; S, 19.70%.

#### 3.2.4 2-(1-(5-Methylthiophen-2-yl)ethylidene)-*N*-(2-morpholinoethyl)hydrazinecarbothioamide (5d)

Yield 77%. Light brown crystalline solid. Mp 156°C–158 °C. FT-IR (ῡ, cm^−1^): 3,312 (N-H), 3,145 (Ar-H), 2,988 (C-H), 1,527 (C=N), 1,109 (C=S); ^1^H NMR (DMSO-*d*_*6*_, 300 MHz) *δ* 2.27 (s, 3H), 2.42–2.45 (m, 7H), 2.53 (t, *J* = 6.3 Hz, 2H), 3.60–3.67 (m, 6H), 6.78 (dd, *J* = 3.6, 0.9 Hz, 1H), 7.28 (d, *J* = 3.6 Hz, 1H), 8.14 (t, *J* = 4.8 Hz, 1H), 10.46 (s, 1H); ^13^C NMR (DMSO-*d*_*6*_, 75 MHz) *δ* 14.6, 15.7, 40.4, 53.5, 56.5, 66.7, 126.6, 128.7, 141.1, 142.7, 145.0, 177.6; LCMS m/z [M + H]^+^: 327.50; Anal. Calcd. for C_14_H_22_N_4_OS_2_: C, 51.50; H, 6.79; N, 17.16; S, 19.64%; Found: C, 51.62; H, 6.91; N, 17.32; S, 19.76%.

#### 3.2.5 2-(1-(3-Chlorothiophen-2-yl)ethylidene)-*N*-(2-morpholinoethyl)hydrazinecarbothioamide (5e)

Yield 65%. Light brown crystalline solid. Mp 116°C–118 °C. FT-IR (ῡ, cm^−1^): 3,321 (N-H), 3,198 (Ar-H), 2,991 (C-H), 1,525 (C=N), 1,105 (C=S); ^1^H NMR (DMSO-*d*_*6*_, 300 MHz) *δ* 2.40–2.42 (m, 7H), 2.53 (t, *J* = 6.3 Hz, 2H), 3.60 (t, *J* = 4.5 Hz, 4H), 3.65 (q, *J* = 5.6 Hz, 2H), 7.13 (d, *J* = 5.4 Hz, 1H), 7.74 (d, *J* = 5.4 Hz, 1H), 8.15 (t, *J* = 4.8 Hz, 1H), 10.64 (s, 1H); ^13^C NMR (DMSO-*d*_*6*_, 75 MHz) *δ* 16.9, 40.6, 53.5, 56.7, 66.7, 123.0, 127.7, 130.5, 135.2, 142.8, 178.0; LCMS m/z [M + H]^+^: 347.40; Anal. Calcd. for C_13_H_19_ClN_4_OS_2_: C, 45.01; H, 5.52; N, 16.15; S, 18.49%; Found: C, 45.09; H, 5.58; N, 16.21; S, 18.61%.

#### 3.2.6 2-(1-(3-Bromothiophen-2-yl)ethylidene)-*N*-(2-morpholinoethyl)hydrazinecarbothioamide (5f)

Yield 80%. Light brown solid. Mp 124°C–126 °C. FT-IR (ῡ, cm^−1^): 3,315 (N-H), 3,097 (Ar-H), 2,983 (C-H), 1,530 (C=N), 1,107 (C=S); ^1^H NMR (DMSO-*d*_*6*_, 300 MHz) *δ* 2.40–2.42 (m, 7H), 2.53 (t, *J* = 6.3 Hz, 2H), 3.59 (t, *J* = 4.5 Hz, 4H), 3.67 (q, *J* = 4.8 Hz, 2H), 7.18 (d, *J* = 5.4 Hz, 1H), 7.73 (d, *J* = 5.1 Hz, 1H), 8.20 (t, *J* = 4.8 Hz, 1H), 10.65 (s, 1H); ^13^C NMR (DMSO-*d*_*6*_, 75 MHz) *δ* 17.3, 40.4, 53.5, 56.8, 66.6, 108.9, 128.4, 133.1, 136.8, 142.8, 178.2; LCMS m/z [M + H]^+^: 391.30; Anal. Calcd. for C_13_H_19_BrN_4_OS_2_: C, 39.90; H, 4.89; N, 14.32; S, 16.39%; Found: C, 40.04; H, 4.97; N, 14.40; S, 16.53%.

#### 3.2.7 2-(1-(5-Chlorothiophen-2-yl)ethylidene)-*N*-(2-morpholinoethyl)hydrazinecarbothioamide (5g)

Yield 90%. Yellow crystalline solid. Mp 148°C–150 °C. FT-IR (ῡ, cm^−1^): 3,321 (N-H), 3,198 (Ar-H), 2,959 (C-H), 1,525 (C=N), 1,105 (C=S); ^1^H NMR (DMSO-*d*_*6*_, 300 MHz) *δ* 2.28 (s, 3H), 2.44 (t, *J* = 4.2 Hz, 4H), 2.53 (t, *J* = 6.3 Hz, 2H), 3.60–3.67 (m, 6H), 7.11 (d, *J* = 3.9 Hz, 1H), 7.35 (d, *J* = 4.2 Hz, 1H), 8.15 (t, *J* = 4.8 Hz, 1H), 10.58 (s, 1H); ^13^C NMR (DMSO-*d*_*6*_, 75 MHz) *δ* 14.1, 40.4, 53.5, 56.5, 66.7, 128.1, 130.9, 142.6, 144.0, 177.7; LCMS m/z [M + H]^+^: 347.40; Anal. Calcd. for C_13_H_19_ClN_4_OS_2_: C, 45.01; H, 5.52; N, 16.15; S, 18.49%; Found: C, 45.13; H, 5.76; N, 16.29; S, 18.63%.

#### 3.2.8 2-(1-(5-Bromothiophen-2-yl)ethylidene)-*N*-(2-morpholinoethyl)hydrazinecarbothioamide (5h)

Yield 61%. Light brown solid. Mp 148°C–150 °C. FT-IR (ῡ, cm^−1^): 3,315 (N-H), 3,146 (Ar-H), 2,965 (C-H), 1,527 (C=N), 1,110 (C=S); ^1^H NMR (DMSO-*d*_*6*_, 300 MHz) *δ* 2.29 (s, 3H), 2.45 (t, *J* = 3.6 Hz, 4H), 2.53 (t, *J* = 6.3 Hz, 2H), 3.60–3.68 (m, 6H), 7.22 (d, *J* = 3.9 Hz, 1H), 7.31 (d, *J* = 3.9 Hz, 1H), 8.16 (t, *J* = 4.5 Hz, 1H), 10.57 (s, 1H); ^13^C NMR (DMSO-*d*_*6*_, 75 MHz) *δ* 14.2, 40.4, 53.5, 56.5, 66.7, 114.7, 128.9, 131.6, 143.9, 145.3, 177.7; LCMS m/z [M + H]^+^: 391.30; Anal. Calcd. for C_13_H_19_BrN_4_OS_2_: C, 39.90; H, 4.89; N, 14.32; S, 16.39%; Found: C, 39.77; H, 4.81; N, 14.30; S, 16.25%.

#### 3.2.9 *N*-(2-morpholinoethyl)-2-(1-(5-nitrothiophen-2-yl)ethylidene)hydrazinecarbothioamide (5i)

Yield 92%. Orange crystalline solid. Mp 192°C–194 °C. FT-IR (ῡ, cm^−1^): 3,317 (N-H), 3,110 (Ar-H), 2,959 (C-H), 1,525 (C=N), 1,107 (C=S); ^1^H NMR (DMSO-*d*_*6*_, 300 MHz) *δ* 2.35 (s, 3H), 2.45 (t, *J* = 4.5 Hz, 4H), 2.55 (t, *J* = 6.0 Hz, 2H), 3.65–3.68 (m, 6H), 7.55 (d, *J* = 4.5 Hz, 1H), 8.09 (d, *J* = 4.5 Hz, 1H), 8.32 (t, *J* = 4.8 Hz, 1H), 10.86 (s, 1H); ^13^C NMR (DMSO-*d*_*6*_, 75 MHz) *δ* 14.2, 40.7, 53.6, 56.5, 66.7, 127.4, 130.9, 142.6, 151.0, 151.1, 177.8; Anal. Calcd. for C_13_H_19_N_5_O_3_S_2_: C, 43.68; H, 5.36; N, 19.59; S, 17.94%; Found: C, 43.82; H, 5.44; N, 19.63; S, 18.00%.

### 3.3 Urease inhibition assay

The indophenol method with a little modification was used to determine the urease inhibitory activity of synthetic compounds (**5a-i**) (Weatherburn, 1967; Yaseen et al., 2016). Thiourea was used as a standard inhibitor. The assay mixture was comprised of 40 μL of buffer (100 mmol/L urea, 0.01 mol/L K_2_HPO_4_, 1 mmol/L EDTA and 0.01 mol/L LiCl_2_, pH 8.2), 10 μL of jack bean urease (5 U/mL) and 10 μL of test compound (1 mM). The reaction mixture was incubated for 30 min with 10 μL urea (1 mM) at 37 °C in 96-well plate. The phenol reagent (40 μL, 1% w/v phenol, 0.005% w/v sodium nitroprusside) and alkali reagent (40 μL, 0.5% w/v NaOH, 0.1% active chloride NaOCl) were added to each well and after 10 min of incubation at 37 °C, the absorbance was measured at 630 nm using a microplate reader (Bio-Tek ELx 800™, Instruments, Inc. United States). The experiments to determine the inhibitory activity of compounds were performed in triplicates. The percentage inhibition was calculated by using the formula given below.

$$\text{Inhibition }\left(\%\right)=100\;–\;\left[\text{Absorbance of the test compound}/\text{Absorbance of the control}\right]\times 100$$

Compounds showing >50% inhibition against urease were further evaluated for the determination of IC_50_ values at various concentrations (0.1, 0.3, 1, 3, 10, 30, 100, 300, 1,000 μM) against jack bean urease. The experiments were performed in triplicate. IC_50_ values were calculated by using the non-linear curve fitting program PRISM 5.0 (GraphPad, San Diego, California, United States) to analyze the results.

### 3.4 Kinetics analysis

The most potent inhibitor **5g** was examined for its mechanism of inhibition against urease by Michaelis-Menten kinetics. The rate of enzyme inhibition was accessed across numerous concentrations of substrate (25, 50, 100, 150 mM) and compound **5g** (0, 1.9, 3.8, 5.7 μM) against urease. Afterwards, the data was plotted to obtain the Lineweaver-Burk plot by using GraphPad Prism version 10.2.1.

### 3.5 *In silico* investigation

#### 3.5.1 Docking studies

For the molecular docking studies, the crystallographic structure of jack bean urease (PDB ID: 3LA4) was retrieved from the RSCB PDB database (Balasubramanian and Ponnuraj, 2010). The structures of compound and enzyme were prepared by protonation with the Protonate3D (Labute, 2007) algorithm implemented in MOE molecular modeling tool (http://www.chemcomp.com/MOEMolecular_Operating_Environment.htm). However, the calculations for the docking investigations were performed using LeadIT from BioSolveIT, GmbH, Germany (www.biosolveit.de/LeadIT). The FlexX application of LeadIT was used for the docking of compounds. Based on the binding free energies, the conformation of the ligand-receptor complex was determined. No modifications were made to the default docking parameters (www.biosolveit.de/LeadIT). The highest affinity for interfacing with the receptor and the most stable poses were determined to possess the lowest free binding energies. Complexes were visualized using Discovery Studio Visualizer v4.

#### 3.5.2 ADMET analysis

An online platform (SwissADME; http://www.swissadme.ch/index.php) was used to determine the pharmacokinetic properties of potent inhibitor **5g**. Several properties such as physical and chemical characteristics, bioavailability, solubility, druglikeness, lipophilicity, pharmacokinetics, and medicinal chemistry of compound **5g** were determined to represent the identified inhibitor as a safer therapeutic agent (Daina et al., 2017). The detailed toxicity analysis was conducted via another user-friendly interface admetSAR (http://lmmd.ecust.edu.cn/admetsar2/). It predicts the hepatotoxicity, carcinogenicity, nephrotoxicity, acute oral toxicity, non-target receptor binding, skin sensitization and ocular sensitivity (Yang et al., 2019). The preADMET (https://preadmet.webservice.bmdrc.org) analysis of **5g** based on pharmacokinetics was also included to validate the predictions. Apart from these software, vNN ADMET (https://vnnadmet.bhsai.org/vnnadmet/login.xhtml) was used which utilizes the vNN approach (a set of 15 predictive models) for ADMET analysis. These models serve as rapid evaluators of crucial characteristics in drug candidates such as drug-related liver injury (Schyman et al., 2017). On the other hand, BioTransformer 3.0 (https://biotransformer.ca) represents a freely accessible online platform designed to integrate machine learning methodologies alongside rule-based systems. The primary objective of this tool is to forecast the metabolism of compounds within various tissues of humans, human gastrointestinal tract and soil and water microbiota from external environment (Wishart et al., 2022). XenoSite (https://xenosite.org) is also an online tool that identifies actual atoms of a drug having probability of transformation during the metabolism in human body (Dang et al., 2020).

#### 3.5.3 Molecular dynamics simulation

The best pose of the docked complex of **5g** was studied using molecular dynamics (MD) analysis based on the docking results. The MD simulations were performed by the iMOD server (http://imods.chaconlab.org/) at 300 K constant temperature and 1 atm constant pressure (López-Blanco et al., 2014). The improved normal mode analysis (NMA) approach in inner coordinates has a user-friendly interface provided by the iMod server. All of the major browsers as well as contemporary mobile devices, are quite receptive and instantaneous with the online interface (Santra and Maiti, 2022).

## 4 Conclusion

In summary, the present work reports the design, synthesis and characterization of morpholine-thiophene hybrid thiosemicarbazones. The structures of these compounds were elucidated by FTIR, ^1^H NMR, ^13^C NMR spectroscopy and mass spectrometry. The synthetic compounds were tested against urease enzyme to combat ureolytic bacterial infections. The evaluation of these compounds against urease revealed a multi-fold superior inhibitory potential compared to the conventional inhibitor, thiourea. Among the tested compounds, **5g** was recognized as the lead inhibitor, exhibiting remarkable inhibitory potential with an IC_50_ value of 3.80 ± 1.9 µM. Enzyme’s kinetics experiments revealed that the potent analogue exhibits uncompetitive inhibition, suggesting a unique mode of action against the urease enzyme. In docking analysis, **5g** displayed a diverse variety of interactions with the active site amino acid residues of urease. *In silico* ADMET profile of **5g** showed the druglikeness and leadlikeness properties with zero violations. Notably, the structural stability of the **5g** complex was demonstrated through molecular dynamics simulations, further supporting its candidacy for preclinical studies. Altogether, the results achieved in the current research work contribute significantly towards the plethora of synthetic inhibitors and could potentially be targeted as a new template for the development of therapeutic candidates for ureolytic bacterial infections.

## Data Availability

The original contributions presented in the study are included in the article/Supplementary Material, further inquiries can be directed to the corresponding authors.
