# Supplementary material for: Exploration of morpholine-thiophene hybrid thiosemicarbazones for the treatment of ureolytic bacterial infections via targeting urease enzyme: Synthesis, biochemical screening and computational analysis
Source: Front Chem. 2024 May 24;12:1403127. doi: 10.3389/fchem.2024.1403127 (PMC11157103; doi:10.3389/fchem.2024.1403127)
Supplement: Supplementary file 1 [file DataSheet1.PDF]

Supplementary Information

Exploration of Morpholine-Thiophene Hybrid Thiosemicarbazones for the Treatment of Ureolytic Bacterial Infections via Targeting Urease Enzyme: Synthesis, Biochemical Screening and Computational Analysis

Rubina Munir[1]*, Sumera Zaib[2]*, Muhammad Zia-ur-Rehman[3], Hira Javed[2], Ayesha Roohi[1], Muhammad Zaheer[3], Nabiha Fatima[1], Mashooq Ahmad Bhat[4], Imtiaz Khan[5]*

[1]Department of Chemistry, Kinnaird College for Women, Lahore, Pakistan

[2]Department of Basic and Applied Chemistry, Faculty of Science and Technology, University of Central Punjab, Lahore, Pakistan

[3]Applied Chemistry Research Centre, PCSIR Laboratories Complex, Lahore, Pakistan

[4]Department of Pharmaceutical Chemistry, College of Pharmacy, King Saud University, Riyadh, Saudi Arabia

[5]Department of Chemistry and Manchester Institute of Biotechnology, The University of Manchester, Manchester, United Kingdom

*** Correspondence:**
Rubina Munir
organist94@gmail.com; rubina.munir@kinnaird.edu.pk

Sumera Zaib
sumera.zaib@ucp.edu.pk

Imtiaz Khan
kimtiaz@hotmail.co.uk

Supplementary Information

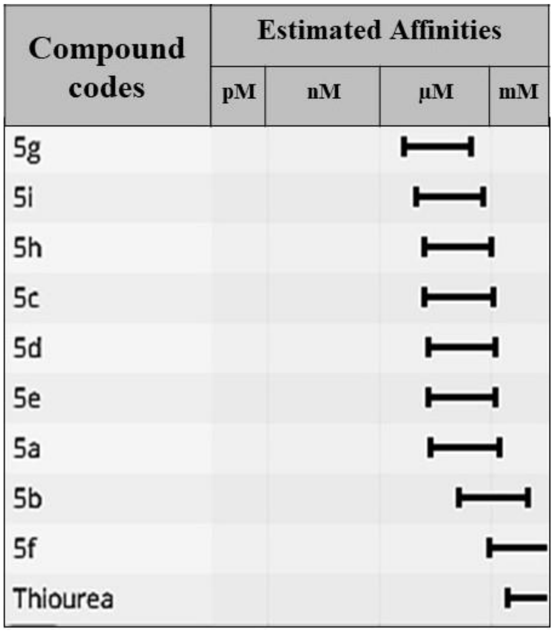

Figure S1: Estimated affinities of compounds

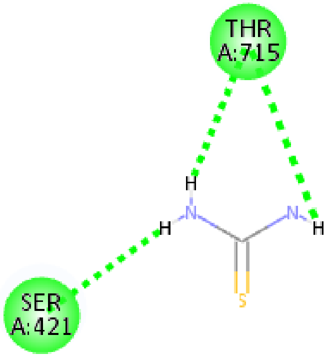

Positive control (thiourea)

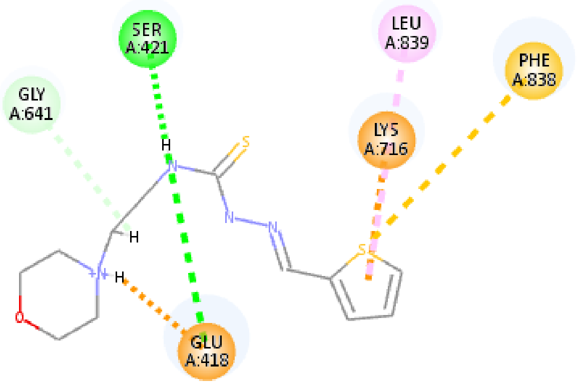

5a

Supplementary Information

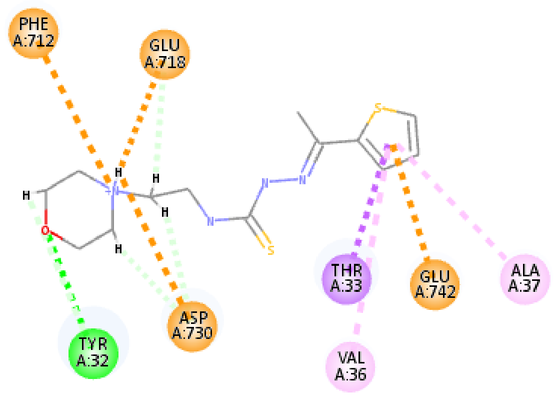

5b

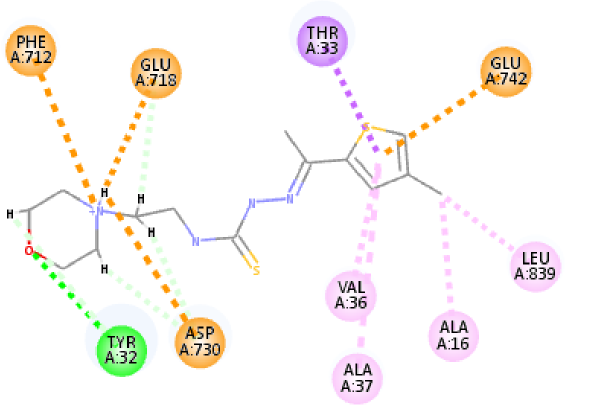

5c

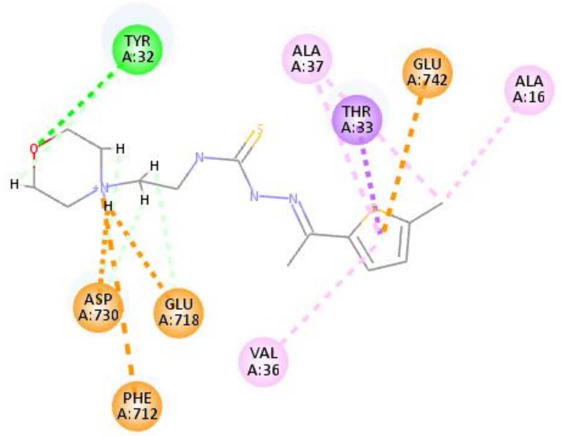

5d

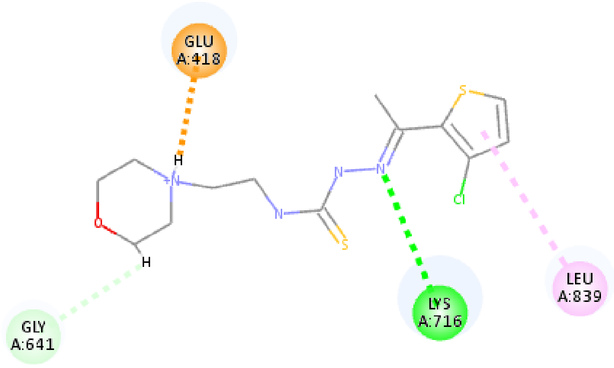

5e

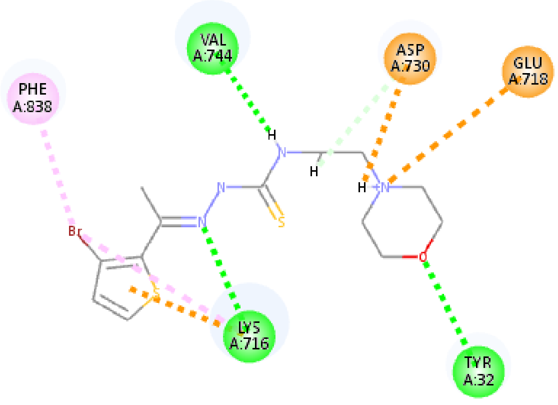

5f

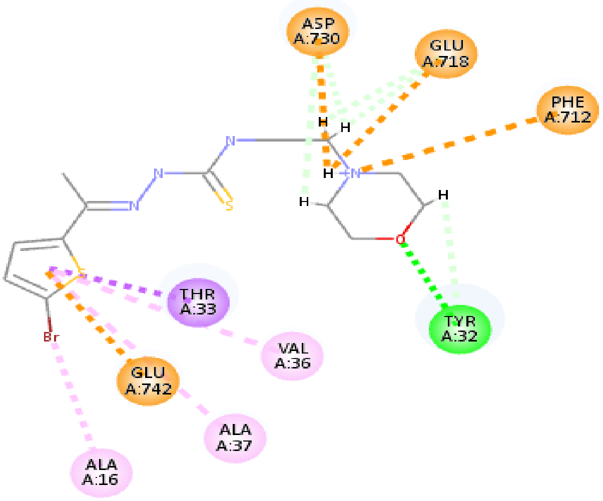

5h

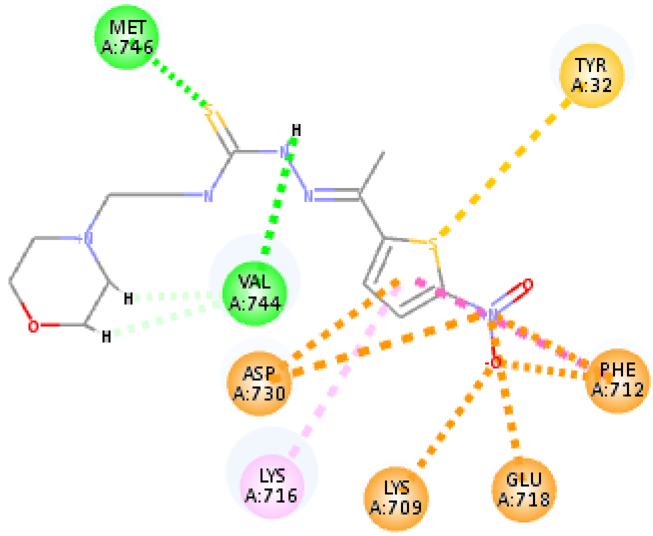

5i

Figure S2: Docking interactions of compounds.
